# Organ Donation and Elective Ventilation: A Necessary Strategy

**DOI:** 10.1155/2017/7518375

**Published:** 2017-01-15

**Authors:** Dolores Escudero, Jesus Otero, Begoña Menéndez de León, Marcos Perez-Basterrechea

**Affiliations:** ^1^Intensive Care Unit, Central University Hospital of Asturias, Oviedo, Spain; ^2^Unit of Transplants, Cell Therapy and Regenerative Medicine, Central University Hospital of Asturias, Oviedo, Spain

## Abstract

Organ transplantation is the sole treatment to improve or save the life of patients with final-stage organ failure. The shortage of available organs for transplantation constitutes a universal problem, estimating that 10% of patients on waiting lists die. Brain death is an undesirable result; nevertheless, it has beneficial side-effects since it is the most frequent source of organs for transplantation. However, this phenomenon is relatively uncommon and has a limited potential. One of the options that focuses on increasing organ donation is to admit patients with catastrophic brain injuries (with a high probability of brain death and nontreatable) to the Intensive Care Unit, with the only purpose of donation. To perform elective nontherapeutic ventilation (ENTV), a patient's anticipated willingness to donate organs and/or explicit acceptance by his/her relatives is required. This process should focus exclusively on those patients with catastrophic brain injuries and imminent risk of death which, due to its acute damage, are not considered treatable. This article defends ENTV as an effective strategy to improve donation rate, analyzing its ethical and legal basis.

## 1. Introduction

Organ transplantation is the sole treatment for improving or saving the life of patients with final-stage organ failure. In 2014, Spain reached a rate of 36 donors per million of the population; despite this, 8–10% of patients on the Spanish waiting list die [1]. According to the World Transplant Registry, at the end of 2014 there were 56,116 European patients waiting for a transplant [2], making patently clear the great imbalance between offer and demand. The shortage of organs is a universal problem and a great worldwide health challenge. In order to reach self-sufficiency, strategies focused on increasing organ procurement, by strengthening the implication of Intensive Care Units (ICUs), are being improved [3].

Brain death (BD) is an undesirable result. However, it has a collateral beneficial effect since it constitutes the most frequent source of organs for transplantation. The problem is that BD is quite unusual and has a limited potential. In recent years, a global drop in BD has been detected related to the decrease of head trauma in traffic accidents, as well as to the improvement in the attention for the neurocritical patient [4]. In Spain, BD represents 2.3% of hospital deaths and 12.4% in ICUs [5].

One of the strategies followed with the objective of increasing organ donation, as well as decreasing the number of patients on waiting lists, consists of admitting patients to the ICU with catastrophic brain injuries and a high probability of evolution to BD.

## 2. Elective Nontherapeutic Ventilation (ENTV)

In 1990, the Royal Devon and Exeter Hospital, in the UK, published a protocol of admission to the ICU of patients with catastrophic brain injury and imminent risk of death, with the exclusive purpose of organ donation [6]. This protocol implied tracheal intubation, mechanic ventilation, and general support until BD had occurred and organ procurement could be arranged. This strategy constituted what is known as ENTV. The authors justified ENTV based on the high mortality of patients on waiting lists, indicating that the insufficient rate of donors per million of the population existing in the UK at that time (14.6) could be increased with this strategy. This work generated a great controversy [7], which concluded in 1994 with the end of its practice by the British Department of Health, due to the absence of a specific legal regulation. Twenty-five years later, ENTV constitutes a habitual clinical practice in US and some European countries, supported by numerous bioethical studies [8–15], and is well accepted by health professionals [12]. Nevertheless, it is not a universal clinical practice yet, generating some ethical and legal controversy in both America and Europe. In recent years, some countries such as the UK, where around 8,000 patients are on the organ transplant waiting list, estimating 1000 deaths per year due to organ shortage [16], claim the necessity of a public discussion about ENTV implantation [16, 17].

## 3. Healthcare Approach

ENTV would be applied to those patients with devastating or catastrophic brain injury, which is defined by the American Neurocritical Care Society as a neurological injury which implies an imminent risk of death and where the treatment of the disease is limited, prioritizing other aspects such as comfort [18]. If the patient had previously expressed willingness to donate organs, or if no evidence of refusal exists, consent for donation will be asked for from relatives. The information must be clear and truthful, indicating that, in order to preserve the organs, tracheal intubation, mechanical ventilation, and admission to the ICU are necessary until BD occurs. The relatives have the option of changing or revoking their decision at any time.

The request of ENTV is a very sensitive issue, especially when circumstances oblige it to be done in the first hours after admission to the Emergency Service. Advanced knowledge in management of neurocritical patients, prognostic factors of evolution to BD, experience in organ donation/transplantation, and training in information management in crisis/request of donation are required. The Transplant Coordinator, which in Spain is mainly a critical care physician, represents the ideal profile for this task. However, if the patient is hospitalized in a hospital without a Transplant Coordinator, or if circumstances require, the request could be realized by other professionals with specific training and a wide knowledge in neurocritical patient management.

## 4. Legislation

The Spanish law 30/1979 about “Organ Procurement and Transplantation” is a law of presumed consent, where it is explicitly indicated that “organ procurement from deceased donors with therapeutic or scientific purposes could be performed if the patient did not express refusal previously” [19]. Nevertheless, in spite of the legal support, this prerogative is not practiced. In Spain, there is no specific regulatory framework for ENTV. It could be interpreted that, bearing in mind the Spanish legislation and in the absence of anticipated willingness from the deceased, clinicians would be backed in performing intubation and admitting the patient to the ICU. Other neighboring countries, which also lack specific legislation for this, defend its practice and consider that it does not infringe the principle of beneficence, while promoting and respecting the beliefs and principles of the patient [9].

## 5. Ethical Considerations

### 5.1. Principle of Beneficence

ICU admission and mechanical ventilation do not violate the principle of beneficence. Even in severe brain injury, some guidelines recommend an intensive treatment during the first 48–72 h to establish a safer prognosis [19, 20]. De Lora and Blanco [8] consider that admission to the ICU is in itself good because it disposes of excellent and plentiful personal and material resources. This allows the establishment of a prognosis and provides the best medical attention, being the place with the best guarantee of supplying the best care, including for those at the end of life. These authors highlight the Bispectral Index Scale of the ICU monitoring systems, which objectively controls and guarantees the adequate sedation of the patient. In these cases, the ICU and the end-of-life care optimize the possibility of a dignified and painless death. In patients which have expressed previously their refusal to be admitted to the ICU (for fear of acquiring a severe disability) and who have manifested their opinion against organ donation, their wishes always prevail and they would never be candidates for ENTV.

### 5.2. Principle of No Maleficence

One of the risks of ENTV is that support treatment can stop brain herniation from occurring. In these cases and in order to preserve the principle of no maleficence, if BD does not occur in a consensus period (usually ≤ 72 h), the clinicians, in agreement with the patient's relatives, would proceed to the withdrawal of all support measures. This would avoid therapeutic futility and the risk of evolution to undesirable clinical situations of severe disability, states of minimal consciousness, or vegetative state. ENTV is supported by numerous bioethical studies [8–15] and in its application and development the necessity of respecting the principles of the patient must always take preference, guaranteeing his/her comfort and absence from pain.

### 5.3. Principle of Justice

Organ donation gives life, representing a public health good. A multicentric study carried out with 1844 BD patients in 42 Spanish hospitals showed that evolution to BD occurs quickly [21]. In those hospitals with Neurosurgery Services, 72% of patients evolved to BD in ≤72 h (48% evolved to BD in the first 24 h). This rate increased in those hospitals without Neurosurgery Services, where 83% of the patients died in the first 72 h, of whom 59% died ≤24 h. Seriously ill patients, with a Glasgow Coma Scale (GCS) of 3–5, evolved to BD mainly in the first 24 hours. Fast evolution to BD, as well as a short ICU stay, is important for the management of the available resources responsibly. Extreme shortage of resources, which could detract attention from other patients with recovery options, could be considered as argument against admission.

### 5.4. Principle of Autonomy

The opinion of the patient about organ donation should be known. If patient's willingness is not known, decisions would be passed to the relatives. The decisions will be based on a patient's preferences, respecting his/her principles, culture, and religion. The choice of donating organs is part of the end-of-life care, constitutes an expression of personal principles, and must be included in the living will.

### 5.5. Affective and Social Management

Information for relatives must be understandable, indicating the extreme gravity and the awful neurologic and vital prognosis, including uncertainty about time needed until BD occurs. The family could be in shock, hampering the adequate assimilation of the information. In these cases, the international recommendations advise stepwise and progressive information in order to allow an adaptation period [19]. Afterwards, when they have been able to take a decision, agreements will be reached by consensus, regarding areas such as the attitude to follow, the maintenance until BD occurs, or the limitation of treatment. The sociocultural differences condition different preferences regarding the kind of medical attention, which explains the great variability in the end-of-life care among different countries [22]. A study realized in US ICUs showed that, indeed in those patients with previous orders of treatment limitation, 23% received cardiopulmonary reanimation while 41% received some kind of vital support [23]. This study indicates the complexity and difficulty of conciliation of the patient's anticipated willingness and clinical decision making in life emergency situations.

**Figure 1:**
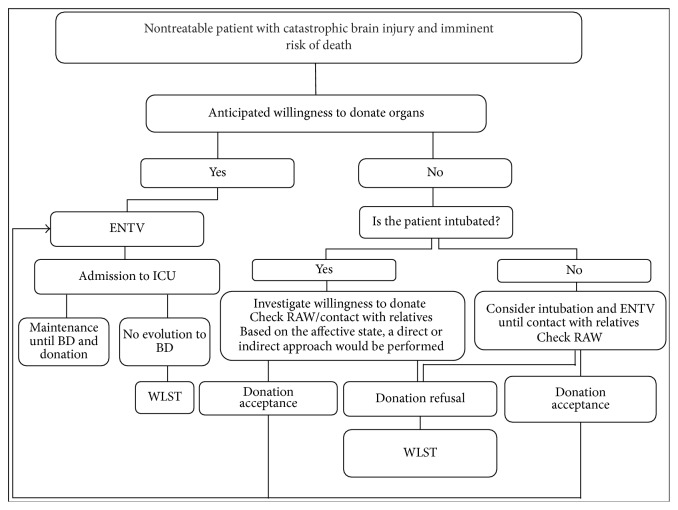
Clinical decision making. ENTV (elective nontherapeutic ventilation), RAW (registry of anticipated willingness), WLST (withdrawal of life-sustaining treatments), BD (brain death), and ICU (Intensive Care Unit).

As with any critical patient, care should be afforded to the relatives of the potential organ donor too [24]. Some studies show that relatives of patients with a catastrophic brain injury have to take up to 60 relevant decisions of an ethical, medical, legal, or financial aspect during the first month of hospitalization, out of which a third have to be taken in the first 24–48 h [25]. Faced with this overburden and depending on the age, personality, profession, and sociocultural condition, patient's relatives can react in a very different way, with an individual approach being essential. Family preferences can change with time, requiring a permanent dialogue and a periodic reevaluation [26]. Doctors must show empathy, create an atmosphere of trust, and provide the information in the right place, setting aside the necessary time, and consider the value that organ donation could represent to their relatives a consolation that partially attenuates their mourning. In the clinical practice, the absence of a patient's anticipated willingness to donate could complicate clinical decision making by both clinicians and the patient's relatives. Nevertheless, a high number of patients arriving to the Emergency Department have received intubation previously by the out-of-hospital Emergency Services. This could facilitate clinical decision making until the patient and relatives' wishes are known (Figure 1).

## 6. Neurological Prognosis

When ENTV is considered, one of the biggest care challenges in clinical practice is to be as accurate as can be with the neurologic prognosis, guaranteeing, as far as possible, that hospitalized patients will evolve to BD. The scales of gravity are useful in order to measure the degree of injury and estimate the probability of death or functional prognosis but are not infallible. The GCS, the Hunt and Hess scale (in subarachnoid hemorrhage), and the intracerebral hemorrhage score [27–30] are more worthwhile when they value cohorts of patients, but they do not guarantee the individual prognosis. In GCS 3, cranioencephalic injury, some authors find up to a 3.6% of acceptable neurologic recovery [27]. Although the probability of error in prognosis is less in cases of an extreme value, it would be difficult to justify at first the end of the treatment knowing that a low percentage can be recovered. Scales of gravity have limitations and their prognostic values improve when they are performed after reanimation. Thus, to minimize the margin of error, periodic repetition over time is recommended, as well as an individual assessment based on personal experience, expectable deficiency by neuroanatomical localization, evolution, comorbidity, and age [26] (Table 1). This global assessment is better than predictive mathematical models, but equally imperfect because it can vary among different doctors. That is why the opinion of an expert multidisciplinary team which minimizes the personal bias and the prognostic error is recommended. Clinical decision making will be based on scientific evidence, clinical experience, and patient's preferences. If uncertainty about prognosis exists, caution will guide clinical decision making. In end-of-life care, error has serious consequences, both in those cases of fast retirement of treatment and in those of overtreatment, provoking an increase of pain, use of unnecessary resources, and high healthcare costs.

## 7. Conclusion

Potential donors are recruited mainly at Emergency, Neurology, and Neurosurgery Services. To perform ENTV, a close collaboration among the implicated services, ICU, and Transplant Coordination Unit is necessary. Anticipated willingness to donate organs of both the patients and their relatives is needed and should focus exclusively on those patients with catastrophic brain injuries and imminent risk of death which are not considered treatable. With the objective of establishing this practice, professionals with an advanced knowledge in neurocritical patient management, as well as a specific training in communication techniques, are required.

## Figures and Tables

**Table 1:** Poor prognostic factors in patients with brain trauma.

Cranioencephalic trauma

High age
Low GCS^a^
Pupillary changes/anisocoria/nonreactive bilateral mydriasis
Alteration or absence of ocular movement
Injuries according to the Marshall CT classification (IV and VI)^b^
Type and gravity of injury according to CT. Presence of traumatic SAH^c^
Intracranial hypertension
Hypoxia/hypotension and presence of secondary injury
Necessity of intubation
Coagulopathy/previous anticoagulant treatment/necessity of blood transfusion
Associated spinal cord injury

SAH

Age
Neurologic function
High score in Hunt and Hess scale
High score in WFNS scale^d^
Fisher scale. Blood volume and location
High score in Ogilvy and Carter scale
Size and localization of aneurism/hemorrhage recurrence
Hyperglycemia

Ischemic and hemorrhagic brain stroke

High score in NIHSS and iScore^e^
High ICH score^f^
Low GCS
Volume of hematoma that varies depending on location:
(i) Basal ganglia hemorrhage ≥ 60 cc, mortality 100%
(ii) Lobar hemorrhage ≥ 60 cc, mortality 71%
(iii) Posterior fossa lethal hemorrhage:
Cerebellar location > 30 cc
Pontine location 5 cc
Diabetes/hyperglycemia on admission
Previous antiaggregation/anticoagulation
Auricular fibrillation

^a^GCS: Glasgow Coma Scale.

^b^CT: computed tomography.

^c^SAH: subarachnoid hemorrhage.

^d^WFNS: World Federation of Neurological Surgeons.

^e^NIHSS: National Institute of Health Stroke Scale.

^f^ICH: intracerebral hemorrhage.
